# Local Validation of a National Orthopaedic Registry

**DOI:** 10.7759/cureus.55636

**Published:** 2024-03-06

**Authors:** Daire-Sean Gibbons, Abdulaziz Mirdad, Lisa Donnelly, Kyra L O'Dwyer, Joy Oguntuase, Aaron A Glynn

**Affiliations:** 1 Regional Orthopaedic Unit, Our Lady's Hospital, Navan, IRL

**Keywords:** trainee, data, validation, registry, arthroplasty, joint

## Abstract

Background/objective: Registries are limited by the quality of the data they collect. We aimed to measure the data entry error rate at a regional orthopaedic unit in a national arthroplasty registry and to assess a proposed intervention of restricting data entry to senior trainees.

Methods and materials: A total of 200 primary and revision arthroplasty cases (119 hips, 81 knees) were randomly selected from a single year, 2020. The Irish National Orthopaedic Registry was examined for the grade of the trainee that populated the form and the accuracy of 24 parameters by comparison with data recorded elsewhere in the patient record.

Results: The mean number of errors per form was 2.17 (95% confidence interval (CI): 1.95-2.39), giving an overall error rate of 9% (95% CI: 8%-10.0%). Eighty-seven percent of forms examined contained inaccuracies, ranging from one to nine errors (4%-38%). Some parameters were more prone to errors, ranging from 1% to 28%. There was no evidence of total errors varying by trainee grade (analysis of variance (ANOVA) p-value: 0.34).

Conclusions: Error rates were in line with the literature. Results did not support restricting data entry to senior trainees.

## Introduction

The first implant registry was established in 1975 in Sweden following suggestions from prominent orthopaedic surgeons, such as John Charnley, that “serious consideration should be given to establishing a Central Register to keep a finger on the pulse of total implant surgery on a nationwide basis” [1]. New technology and information systems have allowed for improvement in the monitoring of implant surgeries for complications, survivability, and other factors [2]. However, registries are limited by the accuracy of the data they collect. The literature on arthroplasty registry data accuracy is scattered and sparse. A focus on ongoing training, education, and feedback on data entry has been shown to reduce errors in other medical contexts [3]. It is likely that, beyond any specific intervention or approach, any initiative that increases staff awareness of the importance of data collection will improve data accuracy. The aim of this validation study was to examine the rates of error in data entry and determine if there is evidence to support a proposed intervention of restricting data entry to more senior trainees. Specifically, we asked the following: 1) What was the error rate in data entry in the Irish National Orthopaedic Registry (INOR) for hip and knee arthroplasty at a regional orthopaedic unit? 2) Are some parameters more prone to error? 3) Do total error rates vary with the seniority of the trainee entering the data? and 4) Do any individual parameter error rates vary with the seniority of the trainee entering the data?

## Materials and methods

A retrospective review of data entry was performed on 200 randomly selected (https://www.random.org/integers/) cases (119 hips, 81 knees). The inclusion criteria were as follows: patients who received unilateral or bilateral elective primary or revision hip or knee arthroplasty at our Regional Orthopaedic Unit during the year 2020. The exclusion criteria were as follows: patients who received emergent arthroplasty secondary to fracture, hemiarthroplasty, resurfacing, and patients operated on outside of the selected timeframe.

**Figure 1:**
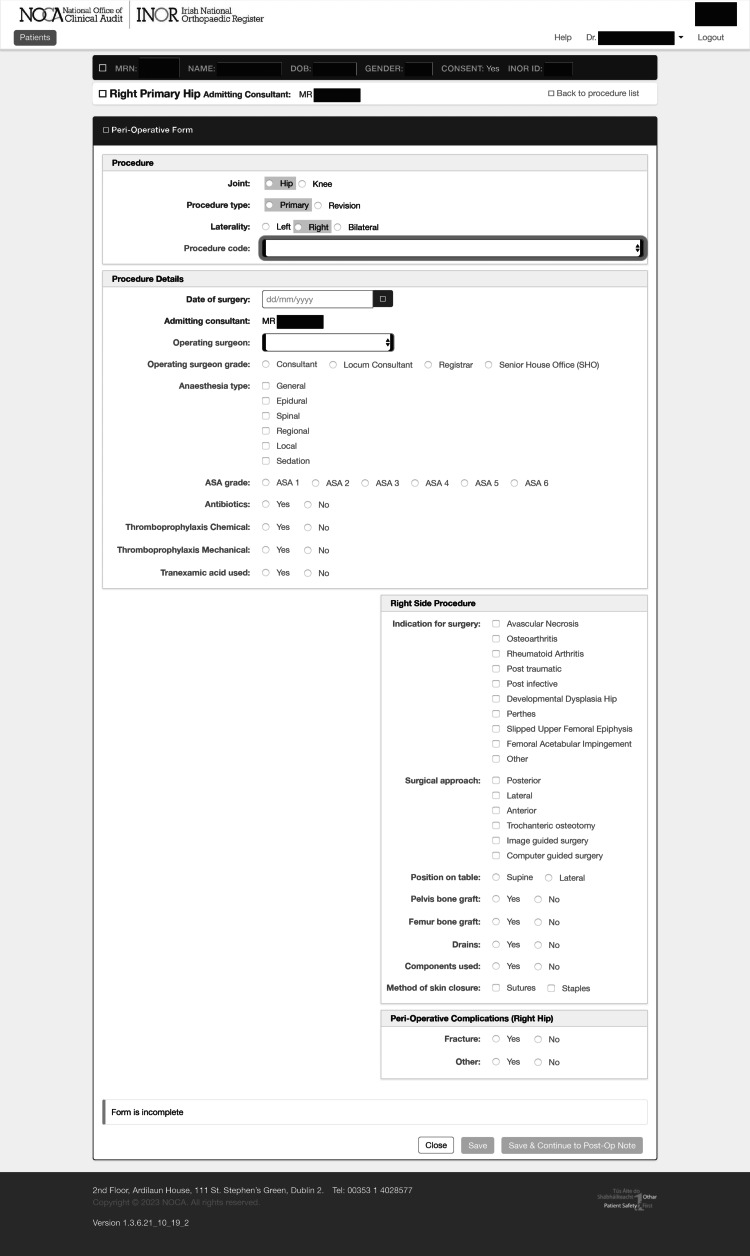
Irish National Orthopaedic Register, excerpt from the web-based user interface. The online portal generates a standardized perioperative form for each arthroplasty case. Patient data and component details are automatically populated from the hospital iPMS system and barcode scanning, respectively. Various other parameters are entered by the user via drop-down menus, tick-boxes, and free-typing.

The INOR utilizes an online web-based user interface (Figure 1) to generate a nationally standardized perioperative form for hip and knee arthroplasty. Primary registry data (i.e., patient and component details) were automatically populated from the hospital-integrated patient management system (iPMS) and theatre barcode scanning. Secondary data (e.g., operative notes, post-op medications, etc.) were entered through a combination of tick-boxes, drop-down menus, and free-typing. This form is completed immediately post-operatively at a dedicated computer station adjacent to the theatre and recovery room. The form was examined for the grade of the trainee who populated the form and the accuracy of 24 data entry parameters. We utilized the “gold standard” manual review of the paper chart, as well as a comparison with the iPMS and the electronic National Integrated Medical Imaging System. This is a well-established method of validating electronically recorded medical data [2,4,5]. It is not possible to complete the form if any parameters are omitted. Categorical parameters were recorded as incorrect if an inappropriate category was selected. Parameters with free typing (Tourniquet time, Op-note components match log, etc.) were considered incorrect if there was any discrepancy. A spreadsheet was generated and examined for trends, and statistical analysis was performed.

Our institution covers a population of 461,000, with a mix of urban and rural areas (52% urban), which is broadly representative of the country (62% urban) [6]. This unit has two dedicated elective theatres operating Monday to Friday, with eight consultant orthopaedic surgeons performing 428 arthroplasties and 1,481 other procedures in 2020 (20% and 10% reduction, respectively, on 2019 due to COVID-19 closure from March 12 to June 8). Data entry was performed by all employed trainees over the year 2020 of the following levels of seniority: 16 Senior House Officers (SHO), junior level trainees with one to three years experience, 102 forms filled; 12 Registrar (Reg), intermediate level trainees with 3+ years experience, 62 forms filled; and five Specialist Registrar (SpR), senior level trainees with 3-8 years experience on a national specialist orthopaedic training scheme, 36 forms filled. As the reviewers were not blinded to the identity of the data recorder or their seniority, a bias may have been introduced. However, in most cases, data entry accuracy was objectively identifiable with minimal subjective interpretation. Any equivocal records due to poor handwriting in paper charts were reviewed by all other reviewers, and a consensus was reached.

Statistical analysis included several approaches. Simple descriptive statistics were used for both the total error rates and the difference in error rates across parameters. The latter was confirmed with the chi-squared test method. The effect of trainee seniority on mean error rates, a continuous variable, was examined with analysis of variance (ANOVA). From a preliminary study, power calculations suggested that only 21 samples per group would be sufficient to detect a difference of one error (4.2% error rate) between groups. Individual parameters were separated, and the error rates for each trainee grade were compared by the chi-squared test with Bonferroni correction rather than ANOVA, to account for parameters with very low error rates (Microsoft Excel for Mac, version 16.66.1).

This study was not subject to a specific ethics board review as it was conducted in line with the Irish Health Information and Quality Authority guidance [7] and the INOR data quality assurance strategy [8].

## Results

The mean number of errors per form was 2.17 (95% confidence interval (CI): 1.95-2.39), giving an overall error rate of 9% (95% CI: 8%-10%). Eighty-seven percent of forms examined contained inaccuracies, ranging from one to nine errors out of 24 parameters examined (4%-38%).

**Figure 2:**
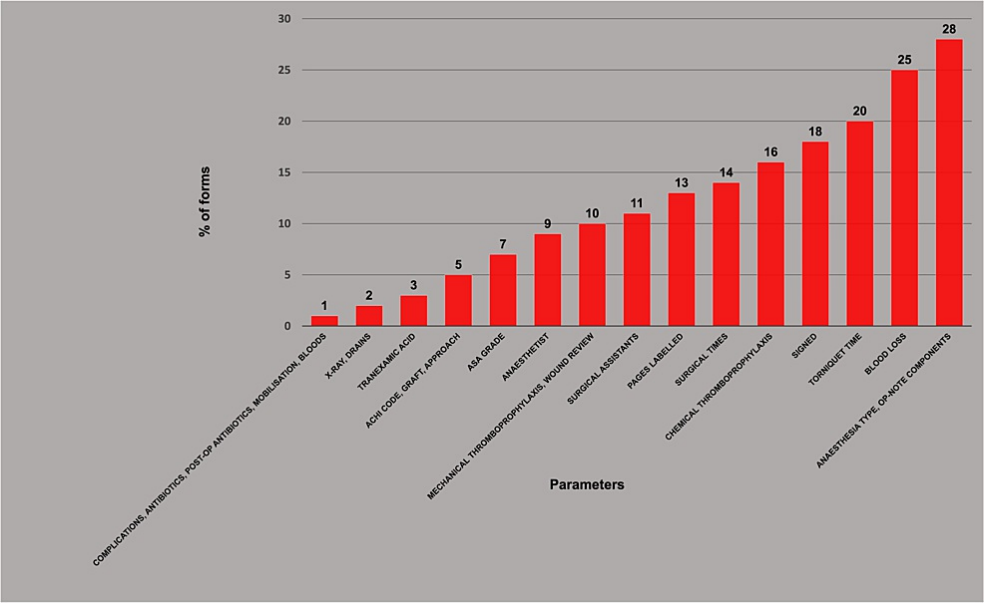
Error rates of individual parameters. Perioperative forms show a wide variation in error rates across the 24 data entry parameters. ASA: American Society of Anesthesiologists

Some parameters were more prone to errors, ranging from 1% to 28%, as shown in Table 1 and Figure 2, suggesting systematic mistakes rather than random typos (chi-squared p-value: <0.01).

**Table 1:** INOR parameter error rates. Twenty-four perioperative form parameters examined for their respective error rates. Analysis of parameter errors by trainee grade for each parameter performed by the chi-squared test with Bonferroni correction identified one parameter with significant differences in error rates between trainee categories* † Tourniquet time only available for data entry on total knee arthroplasies. SHOs: 46, Regs: 22, SpRs: 13. Total: 81 INOR: Irish National Orthopaedic Registry, SHO: Senior House Officer, Reg: Registrar, SpR: Specialist Registrar, Ch-Sq: Chi-squared test, ASA: American Society of Anesthesiologists, ACHI: Australian Classification of Health Interventions

Parameters	Total Errors (%), No. 200	SHO Errors (%), No. 102	Reg Errors (%), No. 62	SpR Errors (%), No. 36	Chi-Sq, p-value
Anaesthesia type	55 (28%)	28 (27%)	14 (23%)	13 (36%)	0.351
Op-note components	55 (28%)	35 (34%)	16 (26%)	4 (11%)	0.026
Blood loss	50 (25%)	30 (29%)	12 (19%)	8 (22%)	0.323
Tourniquet time ^†^	16/81 (20%)	6/46 (13%)	7/22 (32%)	3/13 (23%)	0.181
Signed	36 (18%)	18 (18%)	11 (18%)	7 (19%)	0.969
Chemical thromboprophylaxis	32 (16%)	10 (10%)	13 (21%)	9 (25%)	0.045
Surgical times	27 (14%)	15 (15%)	8 (13%)	4 (11%)	0.851
Pages labelled	25 (13%)	13 (13%)	11 (18%)	3 (3%)	0.097
Surgical assistants	22 (11%)	5 (8%)	9 (15%)	5 (14%)	0.345
Mechanical thromboprophylaxis	20 (10%)	9 (9%)	4 (6%)	7 (19%)	0.101
Wound review	19 (10%)	2 (2%)	13 (21%)	4 (11%)	<0.001*
Anaesthetist	18 (9%)	10 (10%)	6 (10%)	2 (6%)	0.727
ASA grade	13 (7%)	7 (7%)	6 (10%)	0 (0%)	0.169
ACHI code	9 (5%)	6 (6%)	3 (5%)	0 (0%)	0.339
Graft	10 (5%)	4 (4%)	6 (10%)	0 (0%)	0.082
Approach	9 (5%)	4 (4%)	4 (6%)	1 (3%)	0.645
Tranexamic acid	5 (3%)	4 (4%)	0 (0%)	1 (3%)	0.294
X-ray	4 (2%)	1 (1%)	2 (3%)	1 (3%)	0.569
Drains	3 (2%)	1 (1%)	2 (3%)	0 (0%)	0.371
Complications	2 (1%)	1 (1%)	1 (2%)	0 (0%)	0.741
Antibiotics	1 (1%)	0 (0%)	1 (2%)	0 (0%)	0.327
Post-op antibiotics	1 (1%)	0 (0%)	0 (0%)	1 (3%)	0.101
Mobilisation	1 (1%)	1 (1%)	0 (0%)	0 (0%)	0.617
Bloods	1 (1%)	1 (1%)	0 (0%)	0 (0%)	0.617

**Figure 3:**
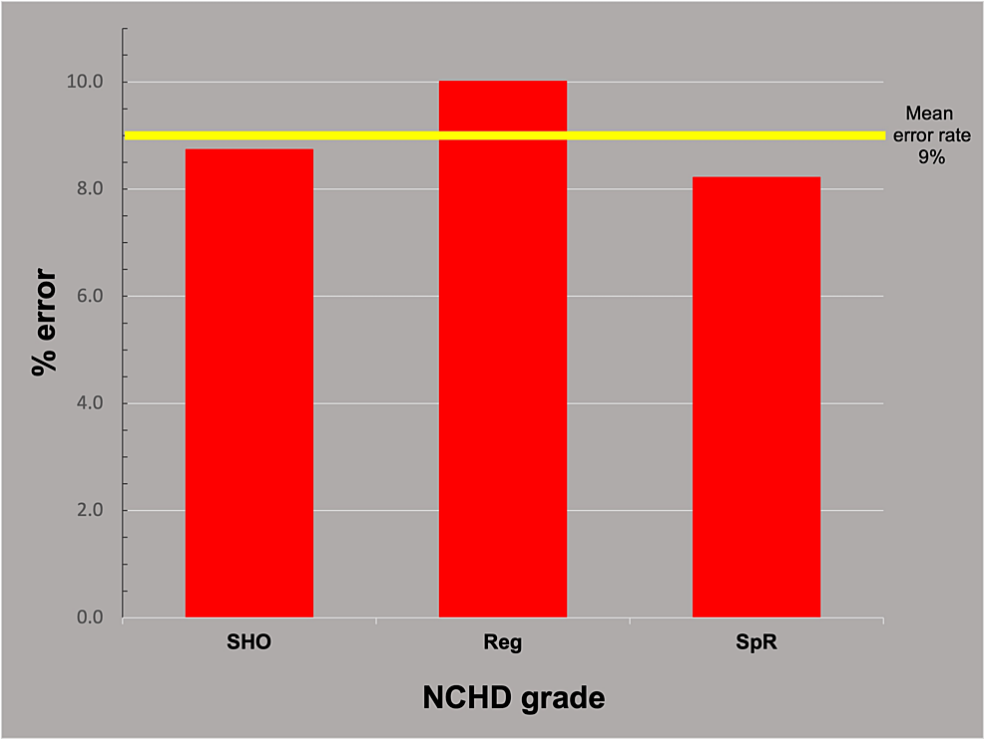
Total errors by trainee grade. The total error rates for trainees of varying seniority show no significant difference. SHO: Senior House Officer, Reg: Registrar, SpR: Specialist Registrar, NCHD: Non-consultant Hospital Doctor

The mean number of total errors did not vary significantly with trainee seniority: combined trainee mean 2.17 (9%), SHO mean 2.10 (8.75%), Reg mean 2.40 (10%), and SpR mean 1.97 (8.2%) (Figure 3) (ANOVA p-value: 0.34.).

**Figure 4:**
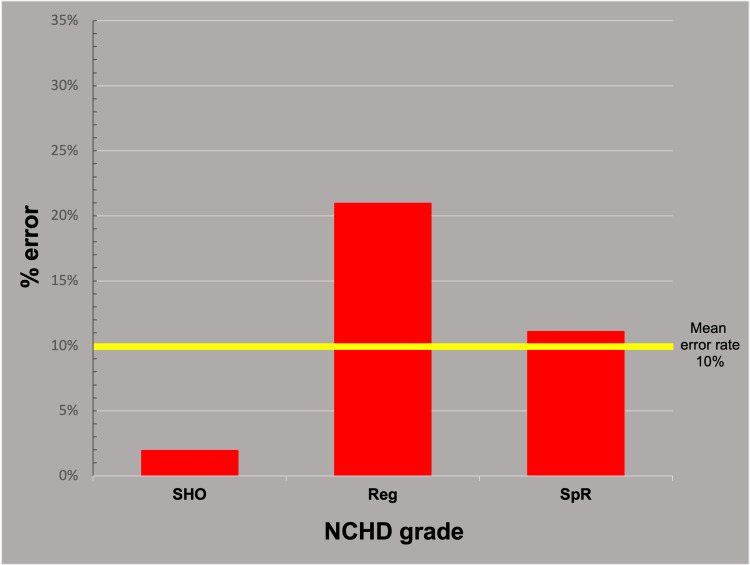
Trainee error rates for the "wound review" parameter. Though statistically significantly different, the error rates for different trainee grades in the parameter "wound review" showed no clear trend. SHO: Senior House Officer, Reg: Registrar, SpR: Specialist Registrar, NCHD: Non-consultant Hospital Doctor, Op-note: Operative-note

The error rate for different trainee grades was calculated for each of the 24 individual parameters recorded (anesthetic type, Op-note components, etc.), and a chi-squared analysis was performed (Table 1). Bonferroni correction suggested that p-values below α=0.0021 should be considered significant. Only one parameter (wound review) met this threshold (p-value: <0.001). However, the error rates for the various trainee grades for this parameter revealed no obvious trend, as illustrated in Figure 4.

## Discussion

The primary goal of arthroplasty registries is to facilitate the recall of substandard devices, by providing a central searchable database that allows clinicians and other parties to quickly identify any patients who received a specific faulty component [1,9]. The secondary goals include facilitating the identification of faulty devices by monitoring results and more generally informing improvements in surgical practice [1,9]. Joint registry studies have many advantages for surgeons, public health, industry, and other interested parties and have shown increasing publication and utility in clinical/planning decision-making [10]. However, all these studies are limited by the accuracy of data entry. Indeed, given that joint arthroplasty is such a successful treatment, and many variations on component design and surgical philosophy exist, the evidence for favoring one component or approach is often marginal. In this scenario, even small systematic errors in data entry may obscure results that high-powered joint registry studies are best placed to address [11].

In many healthcare systems, the responsibility for maintaining patient records is delegated to trainees [12-14]. At our institution, it was proposed that data entry errors might be reduced by restricting data entry to more senior trainees. Though rational [15], there was no firm evidence to suggest that this was the case; hence, it was proposed to conduct a retrospective study to examine our own institution’s error rates and determine if they were higher for more junior trainees. This study is unique in examining the link between data entry accuracy and the clinical role/seniority of the person performing data entry.

The mean number of errors per form was 2.17, giving a total error rate of 9%. Eighty-seven percent of forms were inaccurate, containing between one and nine errors out of a potential 24 data entry parameters. The literature shows wide variability in error rates and a spectrum of various data points being measured in orthopaedic registries [16-19]. In the United Kingdom, the National Joint Registry was compared to provider records and found data missing for between 2% and 8% for hip and knee arthroplasty and up to 21% for ankle arthroplasty [20]. Another study showed a 23% error rate [21]. A study of a single consultant surgeon’s arthroplasty cases in a 12-month period showed that up to 35% of cases had incorrect data inputs [22]. Estimates of general medical registry error rates are equally variable [23-27]. Error rates from this study were moderate and in line with those expected from the literature.

The error rate varied extensively between clinical parameters, ranging from 1% to 28% (p < 0.01) (Table 1, Figure 2). Again, this wide spectrum in the rates of errors between various parameters was expected. For example, the UK National Hip Fracture Database has shown error rates of 7%-42% for various parameters [28]. Random errors would be less likely to affect the results of registry studies as they would be randomly distributed amongst any potential study groups selected, while systematic errors would be more likely to skew the results of registry studies. It is not surprising that parameters with the lowest error rates include the most routine post-op instructions and the most dramatic (e.g., major) complications) [12]. It is equally unsurprising that the parameters with the highest error rates would be the most specific parameters such as tourniquet time, blood loss, and “op-note components match log” [26].

There was no evidence for error rates varying by the seniority of the trainee entering the data (ANOVA p-value: 0.34) (Figure 3). This was not the expected result. The presumption that senior trainees, more familiar with the particularities of arthroplasty, would do a better job [15] was not borne out by the evidence.

Analysis of individual parameters revealed only one parameter (wound review) with significant differences in the rates of errors between trainee grades, after applying the Bonferroni correction for multiple comparisons. No clear trend was observed in this parameter (Figure 4). It is reasonable to conclude that there is little evidence to suggest a strong pattern of variation between error rates of trainees at any level of analysis.

A limitation of this work is that it is a single-institution study, and it would benefit from repetition and validation at other institutions. However, the results shown here should be broadly applicable in other teaching hospitals. Ireland is a small, economically well-developed, European nation, with a population of 5.0 million and Organisation for Economic Co-operation and Development (OECD) demographics comparable with other developed nations: Ireland GDP of $106,852 (ranked 2nd) and income quality index (IEI) of 0.29 (ranked 14th); UK of GDP $49,765 (20th) and IEI of 0.36 (31st); USA GDP of $70,181 (6th) and IEI of 0.38 (33rd); and Germany GDP of $58,784 (14th) and IEI of 0.30 (15th) [29]. The literature confirms that it is common practice in teaching hospitals around the world for trainees to bear a substantial proportion of the burden of medical documentation [12-14]. The finding that error rates for data entry in an orthopaedic registry did not vary with the seniority of the trainee could inform data entry practices in other teaching hospitals. It suggests that data quality improvement measures should be directed at all staff engaged in data entry, rather than restricting data entry to more experienced personnel.

Increasing the accuracy of registry data entry is challenging. Frameworks for data quality improvement in medical registries should be considered during their construction and when validating their performance [23]. One procedure shown to dramatically reduce error rates is intraoperative barcode scanning of components, dropping component errors to 0% [2]. Our own study supports the use of barcode scanning, by showing that free hand recording of “op-note components match log” had the highest error rate of 28%. Technical solutions, such as barcode scanning, are not possible for all data entry parameters, and hence manual data entry is likely essential for the foreseeable future. Familiarity with electronic systems has been shown to rapidly reduce the rates of errors [27], so the use of appropriate introductory training is likely beneficial. Another approach to improving data entry accuracy is the use of two users inputting the same data. Duplicate data entry may flag discrepancies that can be subsequently addressed but also increase the workload of staff. One study of double data vs single data entry found an improvement of 32% in data error rates but was associated with a 37% increase in data entry time [30].

## Conclusions

Arthroplasty registries rely on accurate data entry to support their primary goal of facilitating product recalls and their secondary goal of improving patient care. Little published data exist on evidence-based approaches to improve registry data quality. At our regional orthopaedic unit, we undertook a validation study of error rates and the effect of seniority. Error rates in line with the literature were found. This may affect the ability of the registry to fulfill its secondary objective of identifying risk factors for poor outcomes. Our data would not support a proposed intervention of restricting data entry to more senior trainees.
